# An Atraumatic, Idiopathic Case Report of Intraperitoneal Bladder Dome Rupture

**DOI:** 10.21980/J85S83

**Published:** 2021-10-15

**Authors:** Kylie Prentice, Alisa Wray, Danielle Matonis

**Affiliations:** * University of California, Irvine, Department of Emergency Medicine, Orange, CA

## Abstract

Bladder rupture is a rare condition often seen in trauma patients that is associated with a high mortality.^1^ Spontaneous, intraperitoneal rupture is even rarer in the literature. In this case report we present a middle-aged woman presenting to the emergency department (ED) with abdominal pain, the need to strain to void, and gross hematuria with prior history of urological procedures and cervical cancer. A computed tomography (CT) cystography revealed an intraperitoneal bladder rupture, and the patient underwent an emergent laparotomy. The patient’s postoperative course was uneventful and she was discharged with a Foley catheter. This case represents the importance of keeping bladder rupture in the differential diagnosis even without a history of trauma. Furthermore, an expeditious diagnosis, preferably with CT cystography, and management of the rupture is essential in assuring fortunate outcomes.

**Topics:**

Bladder rupture, urological emergencies, spontaneous bladder rupture.

**Figure:**
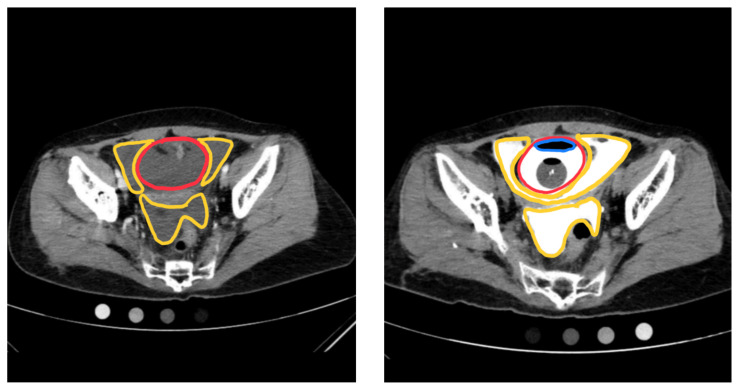
Annotated CT Video Link: https://youtu.be/1nrVhipzC08Unannotated CT Video Link: https://youtu.be/6kE88u7I55ACT Cystography Video Link: https://youtu.be/_uNbLBdYOkE

## Brief introduction

Rupture of the bladder is a rare condition due to the protected location of the bladder within the bony pelvis.^2^ Blunt trauma is the most common cause of bladder rupture, occurring in 60–85% of cases. Blunt trauma resulting in bladder rupture is most commonly due to motor vehicle collisions which can result in pelvic bone fractures.^3^ Penetrating trauma accounts for 15–51% of cases, most commonly due to gunshot wounds. Other etiologies include iatrogenic injuries in the setting of surgery, urological procedures, and even placement of Foley catheters. Spontaneous bladder rupture is an extremely rare condition. Reported etiologies for spontaneous rupture are malignancy, radiation, infection, urinary retention, and vaginal childbirth.^2^

Bladder rupture is associated with a mortality of 22%.^1^ Though this mortality is due mainly to associated severe injuries such as other intra-abdominal organ rupture, severe head injury, and other fractures, delayed diagnosis of the bladder rupture itself is associated with a significantly increased mortality rate.^4^ Therefore, an early and accurate diagnosis is essential for patients. The following is a case report with associated imaging of an idiopathic, intraperitoneal bladder rupture in a female.

## Presenting concerns and clinical findings

The patient is a 40-year-old female with a history of cervical cancer who underwent chemotherapy, radiation, and brachytherapy 8 years prior. Patient was now in remission presenting to the ED with abdominal pain, dysuria, urgency, and the need to strain to void or have a bowel movement. She had previously been seen by urology two months before in the ED with the complaint of gross hematuria with clots. At this prior visit she was found to be in clot retention, had a 24 French 3-way hematuria catheter placed, and admitted to the hospital, where she underwent manual bladder irrigation with large amounts of clots irrigated as well as continuous bladder irrigation (CBI). After her urine was cleared of clots, she was discharged with a Foley catheter and an appointment to see urology for cystoscopy. Unfortunately, she was lost to follow up by urology, and it is unknown when the Foley was removed. At the present ED visit, she was tender on abdominal exam with gross hematuria following Foley placement. Urinalysis was grossly bloody with no leukocyte esterase, nitrites or white blood cells. The patient’s basic metabolic panel, including creatinine, was normal. A CT of the abdomen and pelvis was concerning for nonspecific stranding around the urinary bladder, which radiology stated may be related to her prior cancer treatment versus bladder rupture. A CT cystography revealed an intraperitoneal bladder rupture at the bladder dome.

## Significant findings

On regular CT scan imaging, the urinary bladder is partially distended with contrast with no focal wall thickening or intraluminal hematoma. There is an intraperitoneal bladder rupture with site of rupture likely at the dome of the bladder. The bladder is outlined in red, and the bladder rupture boundaries are outlined in yellow, showing the urine as free fluid escaping into the intraperitoneal space. We also provide these findings in an axial CT in video format. On CT cystography, there is a significant amount of contrast-enhanced urine noted within the visualized peritoneal spaces. The small amount of air present anteriorly is related to the catheterization because a Foley balloon is present within the bladder. These findings are annotated with the peritoneal spaces outlined in yellow, the air in the blue outline, and the bladder in the red outline. All of these CT cystography findings are also presented in an axial view in video format.

## Patient course

With the imaging-confirmed diagnosis of intraperitoneal bladder rupture, urology was consulted, and the patient underwent exploratory laparotomy, and the rupture was repaired. Postoperatively, her course was uneventful. At discharge, she was tolerating a regular diet, ambulating, her pain was controlled on oral medications, and she remained afebrile with normal vital signs. Her Jackson Pratt creatinine value was 0.4 on post-operative day three, and the drain was removed. This value is obtained from testing the creatinine of the fluid from the surgical drain, signifying the absence of surgical site urine leakage. She was discharged home with a Foley catheter for three weeks. Follow up was arranged for her in the urology clinic for a CT cystogram and a voiding trial to assess her post-operative bladder function.

## Discussion

Bladder rupture is relatively rare and occurs most often in cases of blunt trauma, and even then only about 1.6% of patients with blunt abdominal trauma will have a bladder rupture.^2^ Other causes of bladder rupture include iatrogenic injuries in the setting of surgery, urological procedures, placement of Foley catheters and spontaneous bladder rupture due to underlying infection and malignancy.^2^ The rupture can be classified as extraperitoneal, intraperitoneal, and combined extra- and intra-peritoneal ruptures, with these occurring in 63, 32, and 4% of bladder injuries.^5^ The dome is the most likely location of rupture because it is the weakest part of the bladder wall.^5^

Patients with a ruptured bladder can present in a variety of ways, with gross hematuria seen in 67–95% of cases.^3^ Other presentations include sepsis, peritonitis, suprapubic tenderness, difficulty voiding, and low urine output. While X-rays were the traditional way of diagnosing rupture, CT cystography is now commonly used to diagnose this injury because of its rapid turnover time.^3^ An additional advantage of CT cystography is that it can be done simultaneously with a conventional abdominopelvic CT. There are three phases of imaging for a CT cystogram: a non-contrast phase, a contrast phase where water-soluble contrast is administered through a Foley catheter after urine drainage, and a post-void phase. Bladder wall injury may be evident as persistent enhancement in the post-void phase.^6^ Its sensitivity and specificity to detect rupture are reported as 95% and 100%, respectively. Retrograde cystogram can also be used, having similar sensitivities and specificities.^6^

Spontaneous bladder rupture can be the result of a large variety of etiologies; however, the literature consists mainly of case reports. In past literature, spontaneous rupture has been attributed to bladder wall pathology (42%) and retention (35%).^7^ Bladder wall pathology attributed to spontaneous bladder rupture has included diverticulum, tuberculosis, radiation, chronic cystitis, and cancer.^8^ Causes of retention leading to rupture have included bladder outflow obstruction and neurogenic retention. 8 A few cases have reported unique causes, such as an alcoholic binge or after a vaginal delivery in the postpartum period.^8^, ^9^

Extraperitoneal bladder rupture is usually managed conservatively, with Foley catheter drainage and surgery if the injury is not healed in four weeks.^3^ As for intraperitoneal rupture, emergent surgical exploration is the treatment of choice. After repair, the bladder is filled in a retrograde fashion through a urinary catheter to assess for leaks, and an abdominal drain is placed to assess for postoperative leaks. Although there are no strict guidelines for the length of time of the postoperative Foley catheter, the recommended time is 7–14 days.^3^

The patient in this case presented with difficulty voiding, gross hematuria, and a urological history. A speedy and accurate diagnosis of bladder rupture as a result of CT imaging in the ED, with an immediate, successful surgical repair, prevented the fatal complications that are associated with bladder rupture. The etiology of her condition was likely multifocal, possibly due to her recent urological procedures including continuous bladder irrigation, and her prior radiation and brachytherapy for cervical cancer. Bladder rupture, though rare, should be a part of the differential diagnosis even when there is no reported trauma.

## Supplementary Information








