# Supplementary figures and images for: An Atraumatic, Idiopathic Case Report of Intraperitoneal Bladder Dome Rupture
Source: J Educ Teach Emerg Med. 2021 Oct 15;6(4):V9–V11. doi: 10.21980/J85S83 (PMC10332737; doi:10.21980/J85S83)

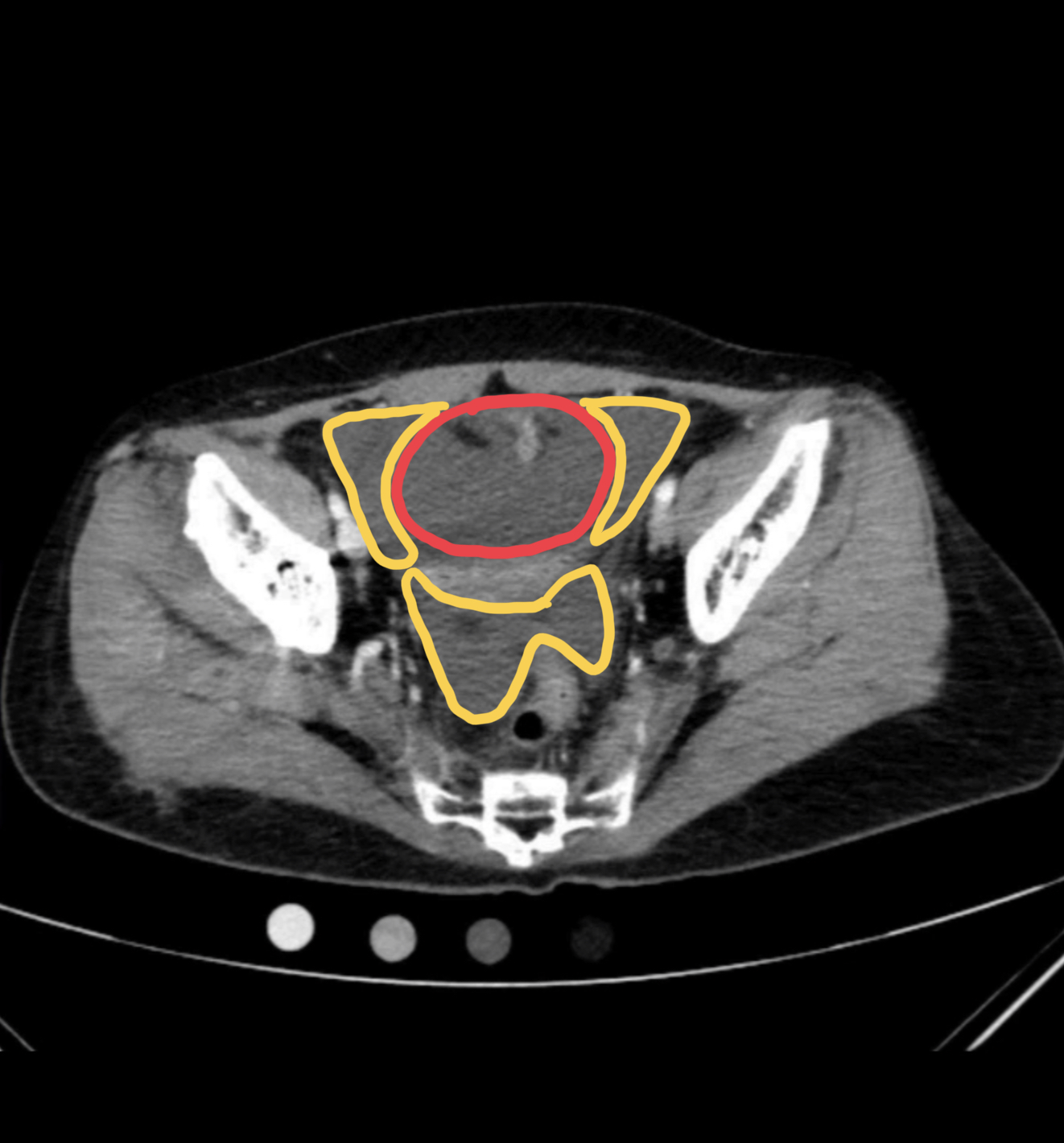

Supplement: Supplementary file 3 [file JETem-6-4-V9-supp3.jpg]

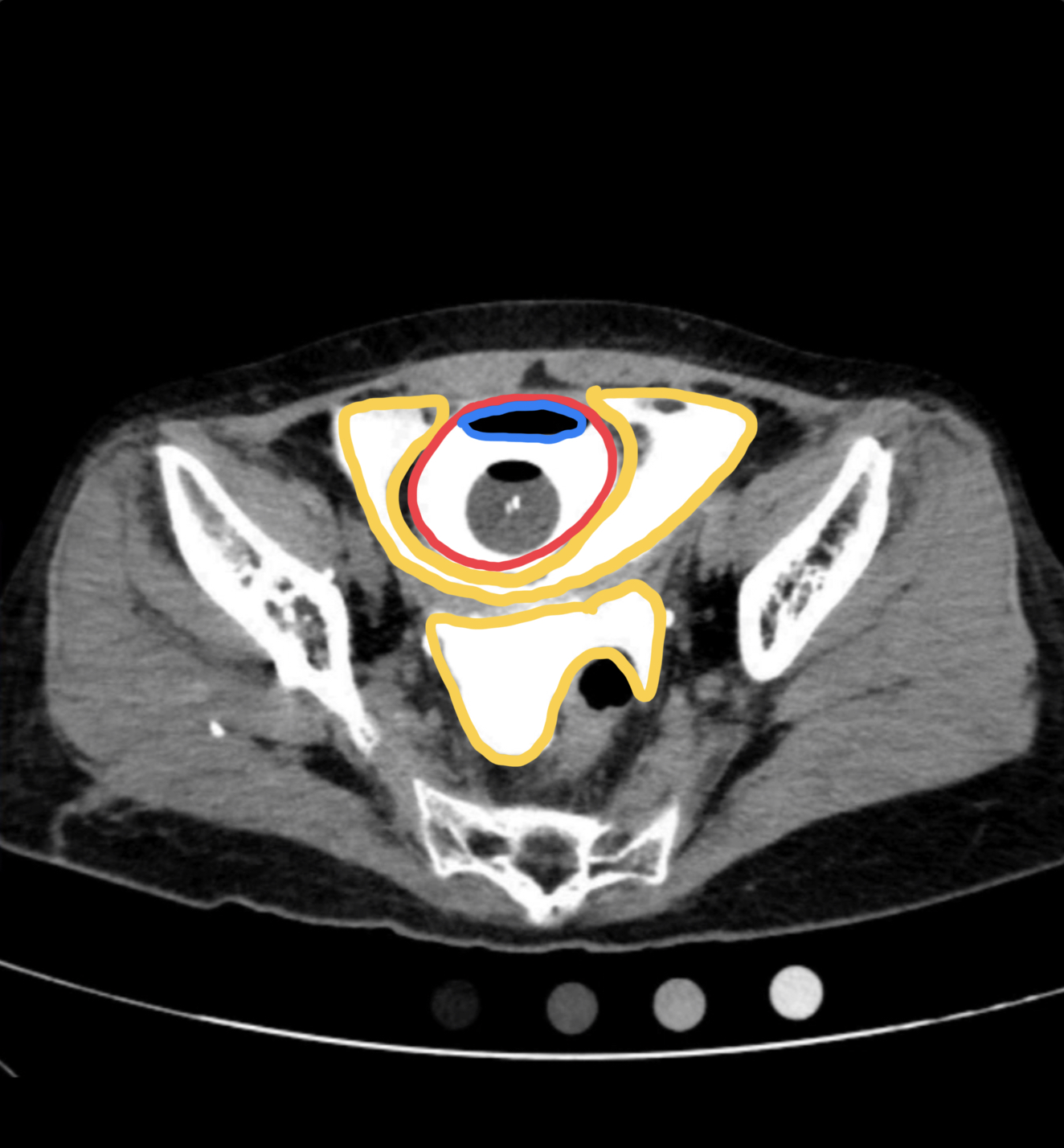

Supplement: Supplementary file 4 [file JETem-6-4-V9-supp4.jpeg]
